# Analysis and prediction of central nervous system tumor burden in China during 1990–2030

**DOI:** 10.1371/journal.pone.0300390

**Published:** 2024-04-17

**Authors:** Zedi Qi, Hongyan Yu, Liangchong Chen, Yichen Qu, Mignda Zhang, Guozhang Qi, Shengli Chen

**Affiliations:** 1 Department of Neurosurgery, The Fifth Clinical Medical College of Shanxi Medical University, Taiyuan City, Shanxi Province, China; 2 Department of Pneumology, The First Affiliated Hospital of Hebei North University, Zhangjiakou City, Hebei Province, China; 3 Department of Neurosurgery, Wenzhou Hospital of Integrated Traditional Chinese and Western Medicine Affiliated to Zhejiang Chinese Medical University, Wenzhou City, Zhejiang Province, China; 4 Department of Neurosurgery, Trigeminal Neuralgia Hospital of Anyang, Anyang City, Henan Province, China; Atal Bihari Vajpayee Institute of Medical Sciences & Dr Ram Manohar Lohia Hospital, INDIA

## Abstract

Central nervous system (CNS) tumors, due to their unique locations, pose a serious threat to human health and present challenges to modern medicine. These tumors exhibit notable epidemiological characteristics across various ethnicities, regions, and age groups. This study investigated the trend of disease burden of CNS tumors in China from 1990–2019 and predicted the incidence and death rate from 2020–2030. Employing data from the 2019 Global Burden of Disease (GBD) database, we utilized key indicators to scrutinize the disease burden associated with CNS tumors in China. The analysis employed the Joinpoint model to track the trend in disease burden, calculating both the annual percentage change (APC) and average annual percentage change (AAPC). Additionally, the Matlab software facilitated the creation of a gray model to forecast the incidence and death rate of CNS tumors in China spanning from 2020 to 2030." In 2019, the age-standardized incidence rate, prevalence rate, death rate, and disability-adjusted life years (DALYs) associated with CNS tumors in China were among the high level in the world. The standardized prevalence rate and DALYs of CNS tumors in China residents showed a stable fluctuation trend with age; however, age-standardized death and incidence rate demonstrated a generally upward trend with age. In China, the age-standardized prevalence and incidence rate of males were lower than those for female residents, while the age-standardized death rate and DALYs among males surpassed those of females. From 1990–2019, the age-standardized prevalence and incidence rate of CNS tumors in China exhibited an increasing trend. The age-standardized death rate and DALYs showed a contrasting trend. According to the gray model’s prediction, incidence rate of CNS tumors would continue rising while the death rate is expected to decline in China from 2020–2023. The burden of CNS tumors in China has shown an upward trajectory, posing significant challenges to their treatment. It is necessary to pay attention to tertiary prevention, start from the perspective of high-risk groups and high-risk factors to reduce the burden of disease, and achieve "early detection, early diagnosis, and early treatment".

## 1 Introduction

Central nervous system (CNS) tumors refer to benign and malignant tumors originating in CNS. Unlike tumors in other systems, the specificity of their onset site determines the higher death and disability rate. The 5-year survival rate of primary CNS tumors is only 1/3 according to the data from the United States, causing severe disease burden every year [1]. There are about 330,000 patients diagnosed as CNS tumors globally every year, while 227,000 patients died for CNS tumors [2]. In 2016, the regions with the highest number of CNS cancer incidence cases were East Asia, followed by Western Europe and South Asia. The countries leading in the most incident cases were China, the USA, and India, ranking in the top three [2]. Similar study also showed that the global death rate of CNS tumors is about 2.8/100000 and 3.2/100000 in China, which is higher than the global average [3]. This phenomenon may be due to the relatively backward level of medical treatment in China in the early days. The data shows that in 2015, brain tumors ranked tenth in the incidence rate of malignant tumors in China. The number of cases was 106,000, with an incidence rate of 7.72 per 100000; the incidence rate and number of cases were higher in urban areas compared to rural areas, and higher in females compared to males [4]. Differences in lifestyles between rural and urban areas and metabolic changes may be responsible for this phenomenon [5]. Therefore, we need to pay more attention to the disease burden of CNS tumors and its changing trend. However, as the country with the largest number of CNS tumors, China lacks in-depth studies on the disease burden of CNS tumors. Based on the information provided by the GBD database, this study analyzed the trend of CNS tumors in China from 1990–2019 and predicted the trend of CNS tumors in China from 2020–2030, aiming to provide a basis for the prevention and treatment of CNS tumors in China.

## 2 Materials and methods

### 2.1 Data sources

The data is from the GBD database, which has been updated to version 2019 and is accessible to users worldwide. The GBD database, created by the Institute for Health Metrics and Evaluation (IHME) at the University of Washington, comprehensively assessed 369 diseases and injury factors in 204 countries and regions worldwide and included the epidemic data of each disease from 1990–2019 (age-standardized incidence, etc.).

### 2.2 Search method

On 1st May, using the GBD-result-tool (https://vizhub.healthdata.org/gbd-results/), we downloaded relevant data, including diseases, gender, region, age, time, study indicators, etc. Subsequently, a clinical physician from our group was responsible for reviewing and conducting a secondary verification. The results were downloaded and saved in a comma-separated values (CSV) format due to its convenience.

### 2.3 Statistical analysis indicators

We gathered data on age-standardized incidence rate, age-standardized prevalence rate, age-standardized death rate, incident cases, prevalent cases, death cases, DALYs, age-standardized DALYs, etc. The "Global Burden of Disease" study, a collaborative effort by the World Health Organization, the World Bank, and Harvard University, introduced DALYs as a comprehensive metric to quantify disease burden. DALYs refer to the total healthy life years lost from onset to death, including the year life lost (YLL) caused by early death and the years lost due to disability (YLD) [6]. This metric enables comparisons of disease burden across diverse regions, independent of population size, and is widely utilized across various fields [7–9].

### 2.4 Research method

Since the GBD database has provided the relevant data and 95% CI, flextable and other packages of R language software (R 4.1.2) can directly adjust the data and form the table. Maps and other packages of R language software can draw a map of the global disease epidemic. Joinpoint regression is an analytical method based on time series data trends, holding significant importance in analyzing changes in disease indicators. By identifying overall trends and inflection points, it allows for a more detailed assessment of trend characteristics in CNS tumor burden indicators across different time periods. After processing the data by the dplyr package, we established Joinpoint regression model by Joinpoint Regression Software (Version 4.9.0.0) to perform the trend analysis and calculated APC and AAPC values (p-values<0.05 were considered statistically significant). APC is used to describe the trend of rate in a certain time period, and AAPC is used to comprehensively evaluate the overall mean change trend of rate covering multiple time periods. The gray model, a model between a white system where the information is completely known and a black system where the information is completely unknown, is suitable for short-term prediction with small data volume. Gray model was first proposed by Professor Deng in 1982 and was widely used since then [10, 11]. To ascertain the feasibility of establishing the GM(1,1) model, it is crucial to ensure that the original data passes the grade ratio test.We performed the model by R Software (Version 4.1.2) to predict the changes of the incidence and death rate of CNS tumors based on the data from GBD by Matlab software. Subsequently, the model needs to undergo relative error test and stage ratio deviation test to ascertain its accuracy. These two tests reflect the deviation between the obtained fitted values and the actual values, expressed as ε(k) and ρ(k) values. Generally, when ε(k) and ρ(k) values are less than 0.1, the model meets high standards.

## 3 Results

### 3.1 Disease burden of CNS tumors in China in 2019

**Fig 1:**
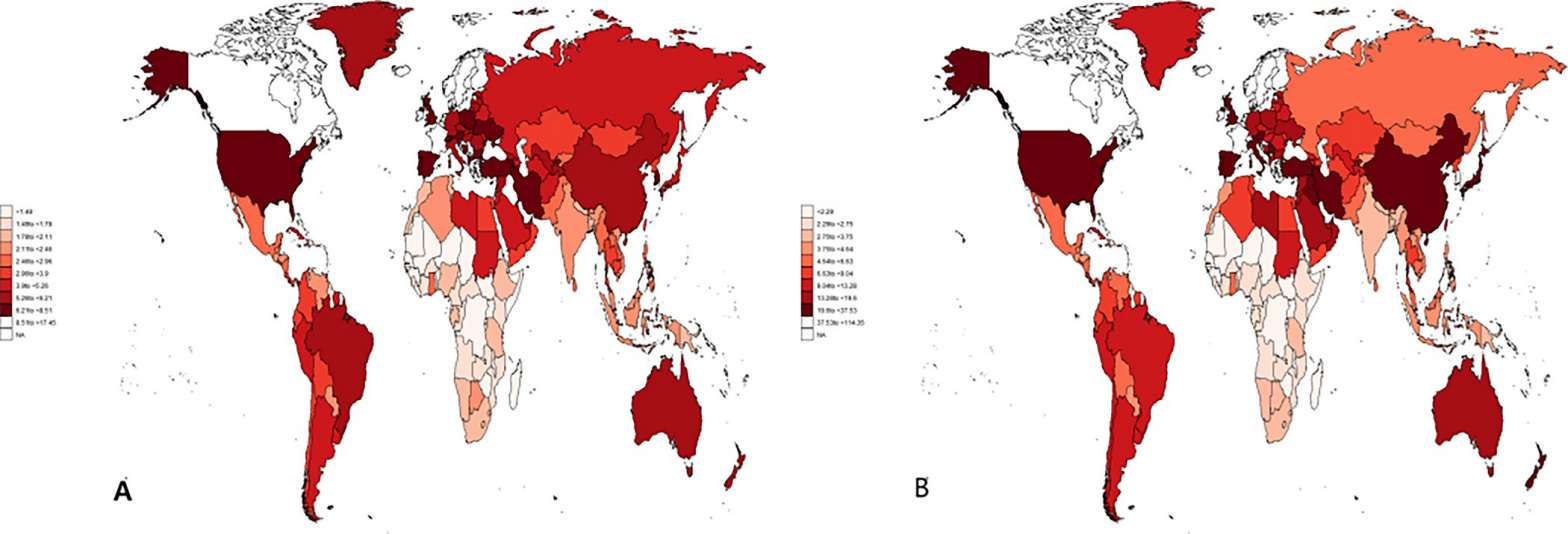
Distribution of age-standardized incidence and prevalence rate (/10^5) of CNS tumors globally. (A and B stand for age-standardized incidence and prevalence rate, respectively).

The GBD database contains the distribution of CNS tumors in 204 countries and regions worldwide. The age-standardized incidence and age-standardized prevalence rate of CNS tumors in China in 2019 were 5.7/100000 (95%CI: 4.4/100000–6.8/100000) and 22.6/100000 (95%CI: 17.4/100000–27.2/100000), ranking 52^nd^ and 36^th^ in the world and higher than most Asian countries. Furthermore, age-standardized death rate and age-standardized DALYs ranked 67^th^ and 68^th^, respectively. Fig 1 shows the distribution of the age-standardized incidence (Fig 1A) and age-standardized prevalence rate (Fig 1B) for CNS tumors in 2019.

### 3.2 Disease burden of CNS tumors in China from 1990–2019

Table 1 shows the disease burden indicators of CNS tumors in China from 1990–2019. In 2019, the prevalence cases of CNS tumors in China was 327890 (95%CI: 256541–399234), which was about 3.6 times the prevalence cases in 1990 (92193 cases). Among them, 129020 males (95%CI:81549–168899) accounted for 39.3%, and 198870 females (95%CI: 144710–269800) accounted for 60.7%. Furthermore, the age-standardized prevalence rate increased from 8.2 / 100000 in 1990 to 22.6/100000 in 2019, with an increase rate of 175.6%. Furthermore, the incidence cases of CNS tumors in China were 94686 (95%CI: 73401–114092), encompassing 47066 males (95%CI: 30531–61735), accounting for 49.7%, and 47620 females (95% CI: 34834–62143), accounting for 50.3%. Moreover, the age-standardized incidence rate escalated from 4.5 per 100,000 in 1990 to 5.7 per 100,000 in 2019, marking a 26.7% increase rate. In contrast, there were varying degrees of alleviation observed in mortality and DALYs. The age-standardized death rate and age-standardized DALYs declined by 10.3% and 21.8%, respectively. Seeing more detailed burden information in Table 1.

**Table 1:** CNS tumors disease burden indicators in China during 1990–2019.

		Year	Male	Female	Total
Prevalence		1990-cases	46394	45799	92193
			(29718–70910)	(28987–61668)	(69713–123623)
		2019-cases	129020	198870	327890
			(81549–168899)	(144710–269800)	(256541–399234)
		Percentage change of cases between 1990 and 2019 (%)	178.1	334.2	255.7
			(53.9–336.4)	(121.9–684.5)	(124.8–394.2)
		1990-rate	8.1	8.3	8.2
		(per 100 000)	(5.2–12.2)	(5.3–11.2)	(6.2–11.0)
		2019-rate	17.6	28.1	22.6
		(per 100 000)	(10.9–23.0)	(20.5–38.0)	(17.4–27.2)
		Percentage change of rate between 1990 and 2019 (%)	117.3	238.6	175.6
			(19.0–242.4)	(67.4–521.0)	(68.7–286.8)
Incidence		1990-cases	25090	20757	45847
			(16495–37767)	(13754–28163)	(35181–61348)
		2019-cases	47066	47620	94686
			(30531–61735)	(34834–62143)	(73401–114092)
		Percentage change of cases between 1990 and 2019 (%)	87.6	129.4	106.5
			(6.0–177.5)	(24.5–274.4)	(39.2–168.5)
		1990-rate	4.8	4.1	4.5
		(per 100 000)	(3.2–7.2)	(2.8–5.6)	(3.5–5.9)
		2019-rate	5.6	5.8	5.7
		(per 100 000)	(3.6–7.3)	(4.3–7.8)	(4.4–6.8)
		Percentage change of rate between 1990 and 2019 (%)	16.7	41.5	26.7
			(∙34.0–68.2)	(∙24.3–132.6)	(∙16.1–64.4)
Deaths		1990-cases	21256	16710	37966
			(14501–31889)	(11635–22365)	(29102–50213)
		2019-cases	35651	27876	63527
			(22009–47443)	(20205–36129)	(47793–76948)
		Percentage change of cases between 1990 and 2019 (%)	67.7	66.8	67.3
			(∙0.2–153.4)	(∙7.3–167.7)	(12.5–117.5)
		1990-rate	4.4	3.4	3.9
		(per 100 000)	(3.0–6.5)	(2.4–3.6)	(3.0–5.1)
		2019-rate	4.1	3.0	3.5
		(per 100 000)	(2.5–5.3)	(2.2–3.9)	(2.6–4.2)
		Percentage change of rate between 1990 and 2019 (%)	∙6.8	∙11.8	∙10.3
			(∙44.6–37.2)	(∙51.3–39.0)	(∙39.6–15.0)
DALYs		1990-cases	1008532	763827	1769659
			(655955–1568959)	(493380–1042817)	(1286809–2432375)
		2019-cases	1178282	875142	2053424
			(743322–1566657)	(649703–1156064)	(1584338–2524972)
		Percentage change of cases between 1990 and 2019 (%)	16.8	14.6	16.0
			(∙32.1–87.8)	(∙40.3–106.0)	(∙23.5–62.7)
		1990-rate	178.2	144.0	161.3
		(per 100 000)	(117.5–276.2)	(93.8–196.0)	(118.0–220.3)
		2019-rate	143.0	109.5	126.2
		(per 100 000)	(89.7–189.0)	(80.8–144.8)	(96.0–154.8)
		Percentage change of rate between 1990 and 2019 (%)	∙19.5	∙24.0	∙21.8
			(∙53.9–27.9)	(∙60.8–38.1)	(∙49.6–9.6)

Note: The 95% CI was written in brackets

**Fig 2:**
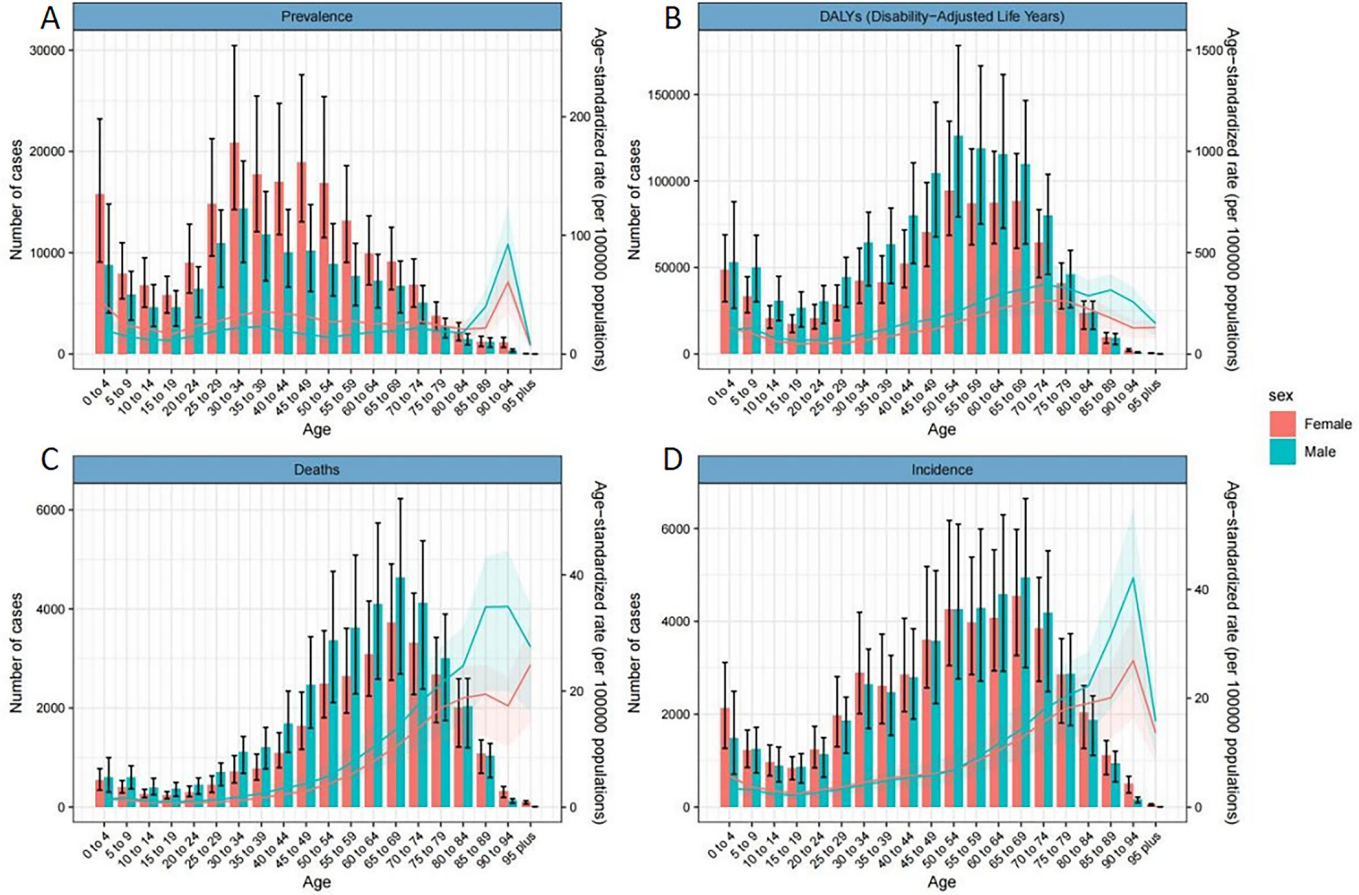
Trends of disease burden indicators of CNS tumors with age in 2019. (A, B, C and D stand for age-standardized prevalence, DALYs, deaths and incidence rate, respectively).

Fig 2 shows the relationship between age and the disease burden indicators. In 2019, the age-standardized prevalence rate among women aged 0–84 years was higher than that of men; however, men exceeded women after the age of 85 years. Furthermore, the highest age-standardized prevalence rate of both men and women was in 90–94 years; the age of 30–34 years had the largest prevalent cases, with women having more patients than men at all ages (Fig 2A). The age-standardized incidence rate was a general upward trend among men and women. Notably, women aged 50–54 years had a higher age-standardized incidence rate than men. However, the data for men was higher after the age of 54 years (Fig 2D). The trend of death cases with age was high in the middle and low at both ends (Fig 2C).

### 3.3 Trend of CNS tumors disease burden in China from 1990–2019

**Fig 3:**
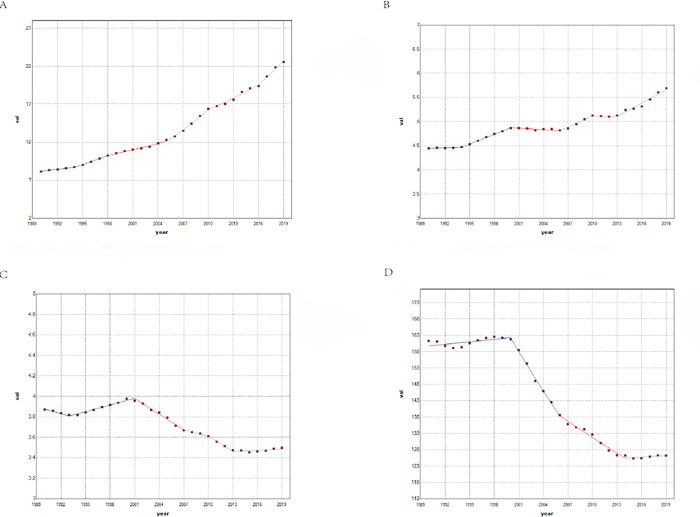
The trend of CNS tumors disease burden with years in China from 1990 to 2019. (A, B, C, and D stand for age-standardized prevalence, incidence, death rate and DALYs, respectively).

From 1990–2019, the prevalent cases, age-standardized prevalence rate, incident cases, and age-standardized incidence rate of CNS tumors in China all showed a continuously increasing trend. The Joinpoint regression model (Fig 3) showed that the age-standardized prevalence rate of CNS tumors in China increased from 1990–2019, with an AAPC value of 3.6 (95%CI:3.3–3.9) and a P-value <0.001; notably, the period of the fastest rise was from 2005–2010, with an APC value of 6.1 (95%CI:5.3–6.8) and a P-value <0.001 (Fig 3A). From 1990–2019, the age-standardized incidence rate generally showed an increasing trend, with an AAPC value of 0.8 (95%CI:0.6–1.1) and a P-value <0.001(Fig 3B); it increased the fastest from 2006–2010, with an APC value of 1.5 (95%CI:0.9–2.2) and a P-value <0.001; however, it decreased from 2000–2006 and 2010–2013 with an APC value <0 and a P-value >0.05. The change in the age-standardized death rate trend was high in the middle and low on both sides, and the AAPC value was -0.4 (95%CI:-0.5–-0.2). In 2001, the age-standardized death rate peaked at 4.0/100000. Subsequently, there was a general downward trend. The decline was the fastest from 2010–2013, with an APC value of -1.4 (95%CI:-2.2–-0.5), and the P-value was 0.004 (Fig 3C). The trend of age-standardized DALYs was similar to the age-standardized death rate, with an AAPC value of -0.8 (95%CI:-0.9–-0.7) and the P-value <0.001. The peak value was 162.5 years in 1998. Subsequently, there was a general downward trend. The decline was the fastest from 2006–2014, with an APC value of -1.2 (95%CI:-1.4–-1.0) and a P-value <0.001 (Fig 3D).

### 3.4 The prediction of incidence and death rate of CNS tumors in China

**Fig 4:**
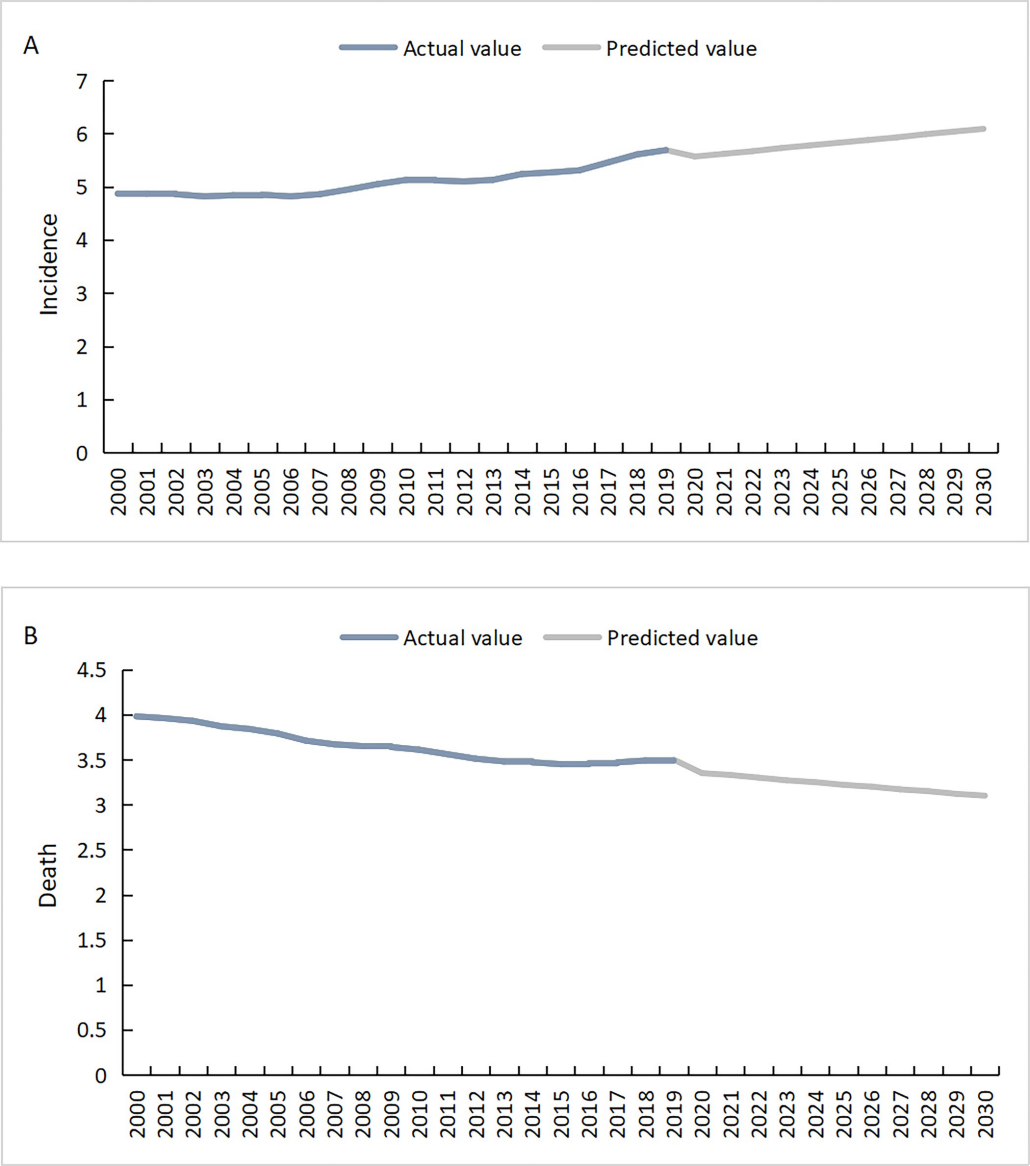
Line diagram of model prediction results of GM. (A and B stand for the incidence and death rate, respectively).

Using the 2000–2019 data as the raw data reference, we constructed gray model by accumulation, fitting, and subtraction using the Matlab software and predicted the incidence and death rate. Before construction, the raw data need to be sure to pass the grade ratio test. The model showed that the incidence rate of CNS tumors in China will increase from 2020–2030, reaching 6.1/100000 people by 2030; however, the death rate will show a downward trend reaching 3.1/100000 by 2030 (Table 2 and Fig 4). Two models passed the relative error test and the stage ratio deviation test,and the ε(k) value and ρ(k) value were less than 0.1.

**Table 2:** The results of gray model.

YEAR	INCIDENCE	DEATH
	RATE(/100000)	RATE(100000)
2020	5.57	3.35
2021	5.62	3.33
2022	5.67	3.3
2023	5.73	3.27
2024	5.78	3.25
2025	5.83	3.22
2026	5.88	3.2
2027	5.93	3.17
2028	5.99	3.15
2029	6.04	3.12
2030	6.09	3.1

## 4 Discussion

In this study, we analyzed the disease burden of CNS tumors with the latest GBD data in 2019, investigated the trends of relevant indicators, and predicted the incidence and death rate. Our findings revealed an upward trend in both age-standardized incidence and age-standardized prevalence rate in China from 1990 to 2019. However, the age-standardized death rate exhibited a consistent downward trend, aligning with present studies [12–14]. However, these findings only focused on different subtypes and specific age ranges of CNS tumors, rather than the overall disease burden changes of CNS tumors. The increase in age-standardized incidence and prevalence rate is partly attributed to the higher disease detection rate caused by economic development and medical technology growth in China, leading to the decline in the age-standardized death rate and DALYs meanwhile. In addition, moderate-high doses of ionizing radiation, certain chemical carcinogens, and tumor-causing viruses may contribute to the increased incidence rate of CNS tumors too. Ionizing radiation has been confirmed as a risk factor for gliomas, meningiomas, and neuroschwannomas, particularly in children [15, 16]. Some carcinogenic chemicals have been found to be potentially associated with the incidence of CNS tumors. An international, multicenter case-control study reported a heightened risk of childhood brain tumors associated with frequent consumption of processed meats during pregnancy, suggesting a potential role of N-nitroso compounds (NOCs) in CNS tumors. However, recall bias among mothers cannot be ruled out [17]. Farmers face a higher risk of brain tumors globally. The relationship between pesticides and brain tumors has been reported [18, 19]. Not only insecticides but also solvents and fertilizers have been confirmed as chemical carcinogens. Air pollution is a challenge faced by most developing countries. It originates from various sources, including industrial emissions, vehicular exhaust, agricultural activities, et al. Currently, there is no conclusive research establishing a direct link between air pollution and brain tumors, but there have been limited exploratory studies [5, 20, 21]. The Chinese Brain "one body and two wings" program has also improved the detection and cure rate of CNS tumors in China. The "two wings" include vigorously strengthening the research on the prevention, diagnosis, and treatment of brain diseases and promoting the study of artificial intelligence through computational and systematic simulation.

Interestingly, males have higher death cases and death rate than females at almost all age groups in our finding. This difference could potentially be attributed to various factors including smoking, alcohol consumption, unhealthy dietary habits, and occupational environments. A case control study from Canada showed that there is no association between smoking and several type of brain tumors [22]. The same conclusion was reached by a meta-analysis [23]. However, smoking habits can pose a significant burden on lung function in CNS tumor patients who underwent brain tumor resection. This might partially explain why the death rate in male patients is higher than in female patients.

According to Fig 1, the CNS tumors disease burdens of the United States, China, Iran, Turkey, Ukraine, Poland, and Australia are at a high level globally. The difference in incidence rate reflects the collective impact of various elements, encompassing genetic predisposition, healthcare access and so on. Prior studies indicate that CNS cancer, particularly glioma, tends to be more prevalent among white populations than among Asian or African populations [24].

The treatment of CNS tumors focus on the combination of multi-subject collaboration cause its high-technique characteristics, including aggressive surgical operation and radiochemotherapy at a suitable time. Additionally, due to its lower incidence rate compared to other tumors, the advanced treatments of CNS tumors are more likely emerge in developed countries and major cities. The current treatments of CNS tumors is still dominated by surgical resection, and the efficacy of chemotherapy and radiotherapy timing, the specific doses, and other new treatments, such as electric field therapy, are still unclear [25]. Therefore, more studies are needed to identify other suitable treatments to reduce the disease burden.

In this study, we predicted the incidence and death rate from 2020–2030 of CNS tumors in China by constructing gray model based on the information extracted from the GBD database. Thus, we observed that the incidence rate of CNS tumors in China showed an upward trend from 2020–2030, while the death rate showed a downward trend. This is related to the growth of medical technology and the recent improvement in people’s health awareness in China. Detection of model indicated that the model accuracy is high and the prediction is accurate.

However, the study still has the following limitations: Firstly, CNS tumor comprises numerous histological types, each with distinct epidemiological characteristics and prognoses. However, this database did not subdivide the CNS accordingly. Moreover, the database only included disease burdens up until 2019, failing to reflect recent changes in the burden of CNS diseases in China. Despite employing models to organize and forecast the data, it remains challenging to totally avoid biases. More analysis and assessment of model accuracy will come with the next update of the GBD database.

In conclusion, despite the decreasing trend of the tumor death rate of CNS tumors in China, the disease burden remains heavy. In this case, more studies and funds should be focused on the treatment of CNS. China needs to increase investment in CNS tumors research, strengthen residents’ health awareness, improve the ability of hospital diagnosis and treatment, put effort into achieving the tertiary prevention-early detection, early diagnosis, and early treatment of CNS tumors simultaneously, especially to strengthen the protection of the elderly and high-risk groups, to reduce the burden of disease.

## List of abbreviations

CSV: comma-separated values
YLD: the years lost due to disability
AAPC: average annual percentage change
APC: annual percentage change
CNS: central nervous system
DALYs: disability-adjusted life years
GBD: Global Burden of Disease
IHME: Institute for Health Metrics and Evaluation
YLL: year life lost
